# Dental Anxiety Prevalence, Correlates, and Patient-Preferred Management Strategies in Romanian Adults: A Cross-Sectional Survey

**DOI:** 10.3390/dj14070438

**Published:** 2026-07-14

**Authors:** Laurențiu Drăguș, Magdalena Rusu-Negraia, Simona-Dana Mitincu-Caramfil, Cătălina-Andreea Florea, Ionela-Violeta Ragea, Valeriu Ardeleanu, Ramona-Oana Roșca, Rares Nicolae Vadana, Marius Moroianu

**Affiliations:** 1 Faculty of Medicine and Pharmacy, Research Centre in the Medical-Pharmaceutical Field, “Dunărea de Jos” University, 800008 Galați, Romania; laurentiu.dragus@ugal.ro (L.D.); ramona.rosca@ugal.ro (R.-O.R.); vadana.rares@yahoo.com (R.N.V.); moroianu.g.marius@gmail.com (M.M.); 2 Faculty of Medicine and Pharmacy, “Dunărea de Jos” University 800008 Galați, Romania; cataflorea2001@yahoo.com (C.-A.F.); iv.dogaru@yahoo.com (I.-V.R.); 3 Faculty of Kinesiotherapy, “Dunărea de Jos” University, 800008 Galați, Romania; valeriu.ardeleanu@gmail.com

**Keywords:** dental anxiety, anxiety-reduction strategies, patient preferences, dentist–patient communication, cross-sectional survey, Romanian adults, dental fear, oral health-related quality of life

## Abstract

**Background:** Dental anxiety constitutes a prevalent barrier to oral healthcare utilization, yet patient-centered data on its prevalence, symptom profile, and preferred management strategies remain scarce in Romanian adult populations. **Objectives:** This study aimed to assess the prevalence and demographic correlates of self-reported dental anxiety, identify the most anxiety-provoking procedures, evaluate the perceived efficacy of anxiety-reduction strategies, and characterize patient preferences for improved anxiety management in the dental setting. **Methods:** A cross-sectional observational study was conducted using an anonymous 18-item online questionnaire (Google Forms) among 223 Romanian adults (≥18 years), recruited via convenience sampling between May and June 2025. Descriptive statistics, Pearson’s chi-square tests, Fisher’s exact test, Cramér’s V, and ordinal logistic regression (proportional odds model) were applied (Python 3.11; pandas, scipy, statsmodels). **Results:** Any degree of self-reported pre-procedural anxiety was endorsed by 60.5% of respondents, with 11.7% reporting moderate-to-severe levels. In the fully adjusted multivariable model (Model 2), female sex (OR = 1.80, 95% CI: 1.04–3.14, *p* = 0.037) and chronic medical conditions (OR = 3.84, 95% CI: 1.21–12.20, *p* = 0.022) were independent predictors of higher anxiety severity, although the chronic disease estimate was based on a small subgroup (*n* = 13) and its wide confidence interval indicates limited precision. Tooth extractions (49.8%) and periodontal surgery (39.5%) were the most anxiety-provoking procedures. Despite this high prevalence of self-reported anxiety, 66.4% of participants had never employed any coping strategy. Detailed procedural explanations provided by the clinician were consistently identified as the dominant patient-preferred intervention across four independent survey items (endorsed by 24.7–77.6% of respondents). A highly significant association was observed between anxiety status and perceived inadequacy of staff anxiety management (χ^2^ = 22.406, *p* < 0.001). **Conclusions:** Self-reported dental anxiety affects the majority of this Romanian adult sample, with communication-based strategies representing the primary patient-centered priority. These findings support integrating structured communication training and standardized anxiety screening into routine dental practice.

## 1. Introduction

### 1.1. Dental Anxiety: Prevalence and Impact on Oral Health

Dental anxiety (DA) represents a significant psychological barrier to oral healthcare worldwide, affecting a substantial proportion of the adult population and posing considerable challenges for both patients and dental practitioners [1,2]. A systematic review and meta-analysis by Silveira et al. (2021), encompassing 31 studies and over 72,000 adults, estimated the global prevalence of dental fear and anxiety at approximately 15.3% (95% CI: 10.2–21.2%), with high dental anxiety reported in 12.4% of individuals and severe forms identified in 3.3% [1]. These figures, while informative, likely underestimate the true burden of the condition, given the variability introduced by different measurement instruments, sampling strategies, and cultural contexts [1,3]. Subgroup analyses have consistently demonstrated a higher prevalence among women and younger adults, suggesting that both sex and age are important correlates of dental anxiety [1,4].

The clinical significance of dental anxiety extends far beyond the psychological discomfort experienced during dental visits. Berggren’s seminal vicious cycle model, proposed in 1984 and subsequently corroborated by multiple empirical studies, describes a self-reinforcing feedback loop in which anxiety leads to avoidance of dental care, which in turn results in deterioration of oral health status, increased need for more invasive and complex treatments, feelings of shame and embarrassment, and ultimately a further exacerbation of the initial anxiety [5,6,7]. Armfield (2013) provided robust cross-sectional evidence for this model, demonstrating that individuals with high dental fear were significantly more likely to delay treatment, present with symptomatic visiting patterns, and report poorer self-rated oral health [6]. More recently, Winkler et al. (2023) confirmed that dental anxiety was significantly associated with reduced adherence to dental care routines and diminished oral health-related quality of life (OHRQoL) in a German adult population [8]. Similarly, Aardal et al. (2023) showed that the behavioral component of dental anxiety, specifically avoidance, was the strongest predictor of impaired OHRQoL, further substantiating the role of avoidance as the central mechanism linking anxiety to adverse oral health outcomes [9].

The downstream consequences of this avoidance pattern are well documented: anxious patients exhibit higher rates of untreated caries, more extensive tooth loss, worse periodontal status, and a greater overall burden of oral disease compared to their non-anxious counterparts [5,7,10]. Furthermore, dental anxiety has been shown to negatively affect broader dimensions of well-being, including sleep quality, social functioning, and general life satisfaction [5,11]. A recent systematic review by Ab Malek et al. (2024), examining the relationship between dental anxiety, dental utilization, and OHRQoL among adults, reinforced these associations, concluding that DA acts as a consistent barrier to regular dental attendance and is inversely correlated with perceived oral health status [12].

From a public health perspective, dental anxiety therefore constitutes not merely an individual psychological challenge but a systemic obstacle to the delivery of effective oral healthcare. Its high prevalence, combined with its detrimental effects on treatment-seeking behavior, oral health outcomes, and quality of life, underscores the urgent need for evidence-based strategies aimed at identifying, preventing, and managing anxiety in the dental setting [2,13]. Understanding the scope and impact of this condition is a prerequisite for designing and implementing targeted interventions that can disrupt the vicious cycle and improve both the experience and outcomes of dental care for anxious patients.

### 1.2. Validated Instruments for Measuring DA

A range of psychometrically validated instruments has supported the assessment of dental anxiety in clinical practice and research, each designed to capture different dimensions of this multifaceted construct. Understanding the strengths and limitations of these tools is essential for contextualizing the methodological choices made in the present study.

The Dental Anxiety Scale (DAS), originally developed by Corah in 1969, is one of the earliest and most widely cited measures of dental anxiety [14]. It consists of four items rated on a five-point scale, with total scores ranging from 4 to 20. While its brevity and simplicity have contributed to its widespread adoption in both epidemiological surveys and clinical settings, the DAS has notable limitations: it does not include an item addressing anxiety related to local anesthetic injections, a major source of fear for many dental patients, and the response options across its four items differ in format and wording, which complicates direct comparisons between items [15,16].

To address these shortcomings, Humphris, Morrison, and Lindsay (1995) developed the Modified Dental Anxiety Scale (MDAS), a five-item instrument with uniform response descriptors scored from 1 (not anxious) to 5 (extremely anxious), yielding a total score of 5 to 25 [15]. The addition of a fifth item assessing anxiety related to local anesthetic injection was a significant improvement, capturing a critical component of treatment-related dental anxiety. The MDAS has demonstrated excellent psychometric properties, including high internal consistency (Cronbach’s α = 0.93), strong test–retest reliability (intraclass correlation coefficient = 0.93), and good discriminant and concurrent validity against the Spielberger State-Trait Anxiety Inventory (STAI) [17]. A score of ≥19 is generally accepted as indicating high dental anxiety or possible dental phobia [15,17]. The MDAS has been translated and validated in numerous languages and cultural contexts, and a systematic survey of its use between 2014 and 2023 identified over 260 published studies employing this instrument, confirming its status as one of the most frequently used dental anxiety measures worldwide [17]. However, considerable variability has been observed in how researchers apply the MDAS, including modifications to cut-off thresholds, response formats, and item wording, which may introduce heterogeneity across studies. More recently, Humphris and Newton (2025) proposed a two-construct formulation of the MDAS, distinguishing between anticipatory dental anxiety (items 1–2) and treatment-related dental anxiety (items 3–5), providing a more nuanced theoretical framework for interpreting the instrument [18].

The Dental Fear Survey (DFS), initially developed by Kleinknecht, Klepac, and Alexander in 1973 and subsequently revised to a 20-item version in 1984, offers a more comprehensive assessment of dental fear [16]. The DFS evaluates three distinct domains: avoidance behavior, physiological arousal during dental treatment, and fear provoked by specific dental stimuli and procedures. Each item is rated on a five-point Likert scale, and the instrument has been shown to have good internal consistency and construct validity across multiple populations [16,18]. While the DFS provides richer clinical information than the DAS or MDAS, its greater length may reduce compliance in survey-based studies.

The State-Trait Anxiety Inventory (STAI), developed by Spielberger, is a widely used general psychological instrument comprising two 20-item subscales measuring state anxiety (a transient emotional response to a specific situation) and trait anxiety (a stable individual predisposition to perceive situations as threatening) [15,19]. The Dental Situation Anxiety Scale (DSAS), a modification of the STAI adapted for dental settings, has also been employed in dental anxiety research [16]. Although the STAI is not specific to dental contexts, it has served as a reference standard for establishing convergent validity of dental anxiety scales, including the MDAS and DFS [19,20].

More recently, the Index of Dental Anxiety and Fear (IDAF-4C+), developed by Armfield in 2010, introduced a multidimensional approach that separately assesses emotional, cognitive, behavioral, and physiological components of dental anxiety, alongside a stimulus module and a phobia screening module [21]. The IDAF-4C+ has been validated in the Romanian language by Done, Preoteasa, and Preoteasa (2023), who demonstrated good internal consistency (Cronbach’s α = 0.945), strong convergent validity with the DAS (r = 0.825), and good test–retest reliability in a sample of 239 adult participants recruited via an online Google Forms questionnaire [21]. Similarly, a Romanian version of the MDAS was validated by Mărginean and Filimon (2012) in a sample of 198 young adults from Western Romania, confirming adequate reliability and positive correlations with the DFS and other psychological measures [22]. Subsequent Romanian studies have successfully employed validated versions of the MDAS and DFS in diverse populations, including school-aged children from central Romania [23], deaf communities from Cluj-Napoca [24], and most recently, dental students from Iași [25].

Despite the availability of these validated instruments in Romanian, the present study employed a purpose-designed, investigator-developed questionnaire rather than a standardized scale. This methodological choice was driven by several considerations. First, the study aimed to explore not only the level of self-reported anxiety but also a broader range of contextual variables that fall outside the scope of existing validated instruments, including specific anxiety-triggering dental procedures, the types of coping strategies previously used and their perceived efficacy, patients’ perceptions of healthcare staff attitudes, and preferences for future improvements in anxiety management within the dental setting. We acknowledge that the MDAS, DFS, and IDAF-4C+ are psychometrically robust instruments; the IDAF-4C+ in particular adopts a multidimensional approach spanning emotional, cognitive, behavioral, and physiological components. These scales were nonetheless designed primarily to quantify anxiety severity and its core components rather than to map the procedure-specific, strategic, and preference-related dimensions targeted here. A methodologically stronger design might have paired a validated severity measure with supplementary purpose-designed items; the use of a single bespoke instrument therefore represents a pragmatic trade-off—prioritizing breadth of patient-centered content and accessibility for online self-administration—rather than a deficiency of the existing scales. This trade-off and its psychometric implications are discussed in Section 4.7.

Second, the questionnaire was designed for online self-administration via Google Forms to maximize accessibility and response rates within the target population, using categorical response options appropriate for a predominantly lay audience. While this approach limits direct numerical comparability with studies employing standardized scales, it provides clinically relevant and ecologically valid data on patient perspectives that can inform practical improvements in dental practice. The limitations associated with using a non-validated instrument are acknowledged and discussed in detail in Section 4.7.

### 1.3. Strategies for Reducing DA: Pharmacological and Non-Pharmacological Approaches

The management of dental anxiety encompasses a spectrum of non-pharmacological and pharmacological interventions, optimally selected according to anxiety severity, patient characteristics, procedural complexity, and available clinical resources [10,13].

Non-pharmacological strategies constitute the first line of anxiety management. Communication-based approaches, including the “tell-show-do” technique and the provision of detailed procedural explanations, have been consistently identified as foundational interventions that reduce patient uncertainty and enhance perceived control [10,13,25]. The quality of the dentist–patient relationship, characterized by empathic communication and active listening, has been shown to influence both immediate anxiety levels and long-term treatment-seeking behavior [10,26].

Relaxation techniques such as diaphragmatic breathing and progressive muscle relaxation have been supported as adjunctive strategies, although the evidence base remains limited to small-scale studies [13].

Cognitive behavioral therapy (CBT), combining psychoeducation, cognitive restructuring, and graded exposure, represents the most extensively studied psychological intervention for dental phobia. Steenen et al. (2024), in a comprehensive meta-analysis of 173 RCTs, found moderate-certainty evidence for CBT in reducing chronic dental trait anxiety (SMD = −0.65, 95% CI [−1.06, −0.24]) [27]; however, its implementation in routine dental practice is limited by the need for specialized training and extended treatment duration [2,28].

Distraction-based modalities have attracted growing research interest. Music listening during dental procedures has been associated with statistically significant reductions in self-reported anxiety in some meta-analyses [29,30,31]. However, the pooled evidence remains heterogeneous and does not yet conclusively support music as an effective stand-alone intervention [27]. Virtual reality (VR) immersion has shown promising results in reducing perceived anxiety and pain [31], with a recent crossover study reporting that VR outperformed nitrous oxide in reducing sympathetic nervous system activity in highly anxious patients [32]. Nevertheless, VR exposure therapy is not yet supported as an effective stand-alone treatment for dental phobia [32]. Other non-pharmacological approaches, including aromatherapy with essential oils and the presence of a trusted companion, have been reported as adjunctive anxiolytic modalities with limited high-quality evidence [10,13].

Pharmacological interventions follow a graduated approach when non-pharmacological methods are insufficient. Oral benzodiazepines provide anxiolysis and amnesia but carry risks of respiratory depression and dependence; Steenen et al. (2024) [27] found low-certainty evidence supporting benzodiazepine-based conscious sedation for reducing state anxiety during oral surgery (SMD = −0.43) Inhalation sedation with nitrous oxide offers rapid-onset, titratable anxiolysis with a favorable safety profile. Intravenous conscious sedation and general anesthesia are reserved for patients with severe phobia or complex procedural needs, requiring advanced monitoring and specialized settings [10,13,33].

Emerging evidence supports an integrated, patient-centered approach combining multiple complementary strategies tailored to individual needs [10,13,33]. However, the patient’s own perceptions of which strategies are most helpful remain underexplored in clinical research focused on objective outcome measures. The present study addresses this gap by directly surveying patients about their experiences, preferences, and recommendations for anxiety reduction in dental settings.

### 1.4. Research Gap and Study Objectives

Despite the growing body of international literature on dental anxiety, research originating from Romania remains limited in scope, sample diversity, and thematic breadth. Existing Romanian studies have made valuable contributions primarily in two areas: the psychometric validation of standardized anxiety instruments and the assessment of dental anxiety prevalence in selected subpopulations. Mărginean and Filimon (2011, 2012) validated the Romanian versions of the MDAS and DFS on a sample of 198 young adults from Western Romania, establishing the linguistic and psychometric suitability of these instruments for Romanian-speaking populations [22,34]. Done, Preoteasa, and Preoteasa (2023) subsequently validated the Romanian IDAF-4C+ on 239 adult participants recruited online from Bucharest, confirming its multidimensional structure and good reliability [21]. Zegan et al. (2019) conducted a cross-sectional study in northeastern Romania with 210 patients using the DAS-R, MDAS, and DCA-R, reporting high dental anxiety in 4.3% and severe anxiety or phobia in 2.9% of the sample [35]. Pacurar et al. (2015) investigated dental fear and anxiety in 406 schoolchildren aged 11–18 from central Romania using the DFS and Dental Beliefs Scale [23], while Suhani et al. (2016) characterized dental anxiety in a deaf population from Cluj-Napoca using the MDAS and DFS [24]. More recently, Armencia et al. (2025) examined the relationship between dental anxiety and quality of life in 180 dental students from Iași, reporting that 6.1% exhibited severe anxiety and that dental anxiety was significantly correlated with impaired physical and psychological quality of life dimensions [36]. Most recently, Moroianu et al. (2026) conducted a cross-sectional case–control study examining the psychosocial impact of dental emergencies in COVID-19 patients, further illustrating the intersection between systemic medical conditions, emergency dental care, and patient psychological burden in a Romanian clinical context [37].

Several critical gaps emerge from this body of evidence. First, existing Romanian studies have focused predominantly on instrument validation, student or pediatric populations, and special needs groups, leaving the general adult population largely unexplored. No large-scale survey has assessed dental anxiety levels and their demographic correlates in a broad sample of Romanian adults attending dental care or recruited from the general public. Second, the existing literature has been almost exclusively centered on quantifying anxiety severity through standardized scales, without investigating the experiential dimensions that are most relevant to clinical practice, namely, which anxiety symptoms patients report, which specific dental procedures they perceive as most anxiety-provoking, what coping strategies they have employed and how effective they considered them, and how they evaluate the role of dental staff in managing their anxiety. Third, there is a notable absence of Romanian data on patient preferences regarding improvements in dental anxiety management, an area that could directly inform the development of context-specific clinical protocols and training programs for dental practitioners. Fourth, to our knowledge, no Romanian study has simultaneously examined all these dimensions within a single integrated survey instrument, making it difficult to draw comprehensive conclusions about the landscape of dental anxiety management from the patient’s perspective. We emphasize that this gap motivates the scope and integrative aim of the present research but does not in itself justify departing from established psychometric standards; the methodological implications of the purpose-designed instrument are considered separately (Section 1.2 and Section 4.7).

The present study was designed to address these gaps by conducting a cross-sectional survey among a broadly recruited sample of Romanian adults, employing a purpose-designed questionnaire that captures multiple complementary domains of the dental anxiety experience. Specifically, the objectives of this study were:

(1) To determine the prevalence and distribution of self-reported pre-procedural anxiety levels among adult dental patients, categorized by demographic characteristics (sex, age group, presence of chronic medical conditions, and frequency of dental visits).

(2) To identify the anxiety symptoms most frequently reported before and during dental procedures, and to determine which types of medical and dental procedures are perceived as most anxiety-provoking.

(3) To assess the types and frequency of anxiety-reduction strategies previously used by patients, and to evaluate which methods are perceived as most effective from the patient’s perspective.

(4) To evaluate patients’ perceptions of dental staff attitudes and behaviors in relation to anxiety management, and to determine which staff-implemented strategies are considered most helpful.

(5) To explore patient preferences and recommendations for improving anxiety management in dental practice, including both non-pharmacological and pharmacological approaches.

(6) To examine bivariate associations between key demographic variables and anxiety levels, use of coping methods, and perceptions of staff attitude, using chi-square analyses.

By integrating these multiple dimensions within a single study, we aim to provide a comprehensive, patient-centered profile of self-reported dental anxiety and its management in a Romanian adult sample, generating data that can inform evidence-based recommendations for clinical practice and dental education in Romania and comparable settings.

## 2. Materials and Methods

### 2.1. Study Design and Ethical Approval

This was a cross-sectional, observational study conducted among Romanian adults (≥18 years), recruited via convenience sampling, investigating the prevalence, correlates, and patient-perceived management of self-reported dental anxiety. The study protocol, including the questionnaire, informed consent form, and data processing plan, was reviewed and approved by the University Ethics Committee (Comisia de Etică Universitară, CEU) of “Dunărea de Jos” University of Galați (Decision No. 21, dated 30 April 2025; file reference HCEU C 3704/29 April 2025). The committee confirmed that the study complied with applicable ethical standards and regulations governing research involving human participants. All procedures were carried out in accordance with the principles of the Declaration of Helsinki (2013 revision) and with the European Union General Data Protection Regulation (GDPR, Regulation 2016/679).

Informed consent was obtained electronically before questionnaire completion. The first page of the online form contained a detailed informed consent statement describing the study’s purpose, the voluntary and anonymous nature of participation, the types of data collected, the intended use of data exclusively for academic research, and the participant’s right to withdraw at any time without consequences or to request modification or deletion of their data. Participants were required to actively select a mandatory consent checkbox confirming that they had read, understood, and agreed to the stated conditions before proceeding with the survey items. Only respondents who provided informed consent were able to access and complete the questionnaire.

### 2.2. Participants and Sampling

The study employed a non-probability convenience sampling strategy. Eligible participants were adults aged 18 years or older, residing in Romania, with sufficient Romanian language proficiency to complete the questionnaire independently. No exclusion criteria based on dental attendance history, medical status, or prior experience with anxiety-reduction methods were applied, in order to capture a broad spectrum of patient perspectives.

Recruitment was conducted entirely online between May and June 2025. The questionnaire was disseminated via social media platforms (Facebook, Instagram, WhatsApp) and direct sharing among personal and professional networks. The Google Forms link was accompanied by a brief explanatory text describing the study’s scope and inviting voluntary participation. A total of 223 valid responses were collected during the recruitment period. No incomplete or duplicate responses were identified, as all items were set as mandatory within the Google Forms platform, and no personally identifiable information (e.g., email addresses or IP addresses) was collected, precluding the identification of duplicate submissions. No a priori sample-size or power calculation was performed, consistent with the exploratory aim of the study; the final sample size (*n* = 223) was determined by the volume of voluntary responses obtained during the recruitment window. As a consequence, several demographic subgroups were small (notably participants aged > 60 years, *n* = 8, and those reporting chronic medical conditions, *n* = 13), and analyses involving these strata are statistically underpowered and yield unstable estimates. These analyses are therefore interpreted with corresponding caution throughout the Results and Discussion (Section 4.7). A post hoc power analysis was not conducted, as observed-power calculations based on the obtained effect sizes provide no additional inferential information.

### 2.3. Survey Instrument

Data were collected using a purpose-designed, self-administered online questionnaire hosted on Google Forms (Google LLC. Google Forms. Available online: https://www.google.com/forms/about/, accessed on 20 April 2025).

The instrument was developed by the principal investigator in consultation with the supervising faculty member, informed by a narrative review of the dental anxiety literature and existing validated instruments (MDAS, DFS, IDAF-4C+), although it does not reproduce or incorporate items from these scales. The questionnaire was designed to capture a broader range of experiential and contextual variables than those assessed by standardized anxiety measures, including specific anxiety-triggering procedures, coping strategies, and their perceived efficacy, perceptions of dental staff attitudes, and patient preferences for improvements in anxiety management.

The questionnaire (Supplementary File S1) comprised 18 items organized into the following thematic domains:

(a) **Demographic and health characteristics** (Items 1–4): sex (male/female), age group (18–30, 31–45, 46–60, over 60 years), presence of self-reported chronic medical conditions (asthma, HIV/AIDS, hepatitis B/C, hypertension, diabetes mellitus; response options: yes/no/not sure), and frequency of medical visits (very rarely, once per year, several times per year, frequently).

(b) **Pre-procedural anxiety level** (Item 5): self-reported anxiety before dental procedures, assessed on a four-point ordinal scale: completely relaxed, slightly worried, very anxious, or extremely anxious with occasional avoidance of procedures.

(c) **Anxiety symptoms** (Item 6): a multi-select item listing six common anxiety symptoms (rapid heart rate, excessive sweating, trembling, rapid breathing/difficulty breathing, intense negative thoughts, avoidance of procedures due to fear), with respondents instructed to select all applicable options.

(d) **Anxiety-provoking procedures** (Items 7–8): the type of medical procedure causing the most stress or anxiety (Item 7: blood tests/injections, dental procedures, surgical interventions, imaging investigations, general medical consultations; single-choice) and the specific dental procedure perceived as most anxiety-provoking (Item 8: simple or complex dental extractions, periodontal surgery, caries treatment, professional cleaning; single-choice).

(e) **Anxiety-reduction methods used and perceived efficacy** (Items 9–11): prior use of any anxiety-reduction method (yes/no/not sure; Item 9), specific methods used (multi-select; Item 10: breathing/relaxation techniques, music/distraction, cognitive-behavioral therapy, companion support, detailed explanations from the clinician, anxiolytic medication, none), and the single method perceived as most effective (Item 11; single-choice with the same categories plus a “none” option).

(f) **Perception of healthcare staff attitudes and strategies** (Items 12–13): characterization of staff attitude toward anxiety management (very empathic and supportive, professional but distant, inappropriate; Item 12; single-choice), and the staff-implemented strategy considered most helpful (detailed procedural explanations, creating a relaxing environment, pauses during the procedure, emotional support through a calm and empathic demeanor; Item 13; single-choice).

(g) **Post-strategy confidence and perceived adequacy of anxiety management** (Items 14–15): self-reported confidence in future procedures after applying anxiety-reduction strategies (Item 14; four-point ordinal scale) and whether the respondent had ever felt that their anxiety was inadequately managed by healthcare staff (Item 15; four response options).

(h) **Patient preferences for improvement** (Items 16–18): desired improvements in clinical practice for anxious patients (Item 16; single-choice among four options), strategies considered most effective for reducing dental anxiety (Item 17; multi-select with nine options including detailed clinician explanations, deep breathing techniques, music listening, additional local anesthesia, distraction methods, mild sedation, emotional support from a companion, aromatherapy, and caffeine avoidance), and a single best recommendation for improving dental care regarding anxiety (Item 18; single-choice among nine options).

All items used categorical response formats (single-choice or multi-select checkboxes). No open-ended questions were included. Item content was informed by a narrative review of the dental anxiety literature and of existing validated instruments (MDAS, DFS, IDAF-4C+), and the draft questionnaire was reviewed by the supervising faculty member prior to deployment; this constituted a limited form of expert content review rather than a formal, multi-expert content validity assessment. The questionnaire was subsequently pilot-tested informally among five adult volunteers to assess clarity, comprehensibility, and completion time (estimated at 5–7 min), and minor wording adjustments were made based on their feedback. It should be emphasized that no cognitive interviewing, structured expert-panel evaluation, quantitative content-validity procedure (e.g., content validity index), or formal psychometric validation (test–retest reliability, internal consistency, or criterion validity) was performed. The absence of these procedures is a substantive methodological limitation, given that the study’s contribution rests on an investigator-designed instrument; accordingly, the present findings should be regarded as exploratory and hypothesis-generating and require confirmation using a psychometrically validated tool (see Section 4.7).

### 2.4. Statistical Analysis

All statistical analyses were performed using Python (version 3.11; Python Software Foundation) with the pandas (version 2.1), scipy (version 1.11), and statsmodels (version 0.14) libraries. Descriptive statistics were used to summarize demographic characteristics and questionnaire responses, expressed as absolute frequencies (*n*) and percentages (%). For multi-selected items, percentages were calculated relative to the total number of respondents (*n* = 223), as each participant could select multiple options.

Bivariate associations between categorical variables were assessed using Pearson’s chi-square (χ^2^) test of independence. Specifically, chi-square analyses were conducted to examine associations between demographic variables (sex, age group, presence of chronic conditions, and frequency of medical visits) and the following outcome variables: self-reported anxiety level (Item 5), prior use of anxiety-reduction methods (Item 9), and perception of staff attitude (Item 12). When more than 20% of expected cell frequencies were below 5, Fisher’s exact test was used as an alternative. The threshold for statistical significance was set at *p* < 0.05 (two-tailed) for all analyses.

To further explore independent associations between demographic predictors and anxiety level, an ordinal logistic regression (proportional odds model) was planned, with the four-level anxiety variable (Item 5) as the dependent variable and sex, age group, and chronic disease status as independent predictors. The proportional odds assumption was formally evaluated using the Brant test (both the omnibus test and per-predictor tests); a multinomial logistic regression was pre-specified as an alternative in case of violation. As a sensitivity analysis, the Brant test was repeated on a three-level version of the outcome in which the two most severe categories were merged, to verify robustness given the small cell count in the extreme-anxiety category. Given the exploratory nature of the study and the use of a non-validated instrument, no formal adjustment for multiple comparisons was applied to the primary analyses, in order to avoid the inflation of Type II error that aggressive correction would entail in a hypothesis-generating context. However, to allow transparent interpretation, we note that twelve bivariate chi-square tests were conducted, corresponding to a Bonferroni-corrected significance threshold of α = 0.05/12 = 0.0042. Associations with *p*-values above this threshold but below 0.05 are accordingly interpreted as tentative and in need of confirmation in adequately powered studies (see Section 3.2).

## 3. Results

### 3.1. Demographic Characteristics

A total of 223 adults completed the online questionnaire. All participants provided informed consent before participation. The sociodemographic characteristics of the study sample are summarized in Table 1.

The majority of respondents were female (*n* = 153; 68.6%), while males constituted 31.4% of the sample (*n* = 70). Regarding age distribution, the largest group comprised participants aged 18–30 years (*n* = 139; 62.3%), followed by the 31–45 age group (*n* = 48; 21.5%), the 46–60 age group (*n* = 28; 12.6%), and participants older than 60 years (*n* = 8; 3.6%). The sample thus exhibited a marked predominance of younger adults, consistent with the online recruitment strategy employed.

The vast majority of participants reported no chronic medical conditions (*n* = 206; 92.4%), while 13 respondents (5.8%) reported having at least one chronic disease (including asthma, hypertension, diabetes mellitus, or viral hepatitis), and 4 participants (1.8%) were unsure of their chronic disease status.

With respect to the frequency of dental visits, nearly half of the participants reported attending dental care once per year (*n* = 106; 47.5%). Approximately one quarter visited the dentist several times per year (*n* = 59; 26.5%), while 22.9% (*n* = 51) reported very infrequent attendance (once every few years). Only a small proportion of respondents (*n* = 7; 3.1%) reported frequent visits on a monthly or weekly basis. Overall, 70.4% of the samples reported attending dental care at least once annually, whereas 22.9% exhibited patterns of irregular or infrequent dental attendance.

### 3.2. Pre-Procedural Anxiety Levels

**Figure 1:**
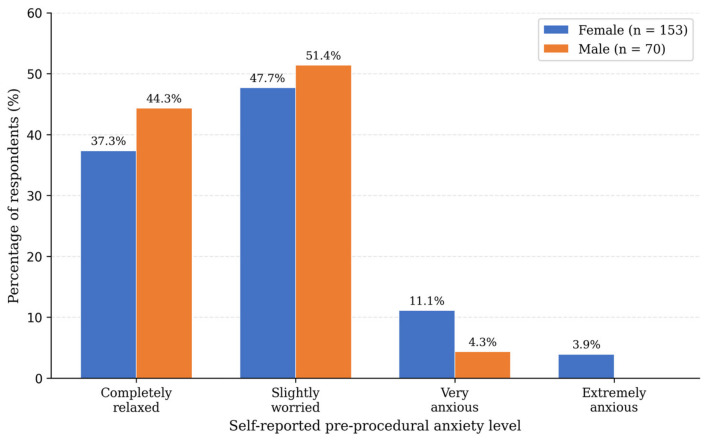
Distribution of self-reported pre-procedural dental anxiety levels by sex (*n* = 223). Bars represent the percentage of respondents within each sex category. Anxiety levels were self-reported using a four-point categorical scale.

The distribution of self-reported pre-procedural anxiety levels is presented in Table 2 and Figure 1. When asked about their general emotional state before a dental procedure, approximately two-fifths of participants (*n* = 88; 39.5%) reported feeling completely relaxed, while nearly half (*n* = 109; 48.9%) described themselves as slightly worried. A smaller proportion reported higher levels of anxiety: 20 respondents (9.0%) indicated feeling very anxious, and 6 (2.7%) reported being extremely anxious, with some acknowledging avoidance of dental procedures altogether. Thus, the combined prevalence of any degree of self-reported pre-procedural anxiety in this sample was 60.5% (*n* = 135), whereas moderate-to-severe anxiety (defined as very anxious or extremely anxious) was reported by 11.7% of participants (*n* = 26).

When stratified by sex (Table 2; Figure 1), female participants exhibited a numerically higher prevalence of elevated anxiety compared to males. Among women, 37.3% reported feeling completely relaxed, 47.7% were slightly worried, 11.1% were very anxious, and 3.9% were extremely anxious. In contrast, 44.3% of male respondents reported being completely relaxed, 51.4% were slightly worried, and only 4.3% reported being very anxious, with no male participant endorsing the extreme anxiety category. The overall distribution of anxiety levels did not differ statistically significantly between sexes (χ^2^ = 5.977, df = 3, *p* = 0.113). However, when anxiety was dichotomized into moderate-to-severe (very anxious or extremely anxious) versus low or no anxiety, a statistically significant difference emerged (Fisher’s exact test, *p* = 0.023; OR = 3.96, 95% CI: 1.14–13.76), indicating that female participants were significantly more likely than males to report moderate-to-severe pre-procedural dental anxiety.

Regarding age distribution (Table 2), the proportion of completely relaxed participants was relatively stable across age groups, ranging from 35.7% in the 46–60 group to 47.9% in the 31–45 group. The 18–30 age group, which constituted the majority of the sample, showed that 37.4% were completely relaxed and 51.1% were slightly worried. Notably, the >60 age group exhibited the highest proportion of very anxious respondents (25.0%), although this subgroup was small (*n* = 8). No statistically significant association was found between age group and anxiety level (χ^2^ = 5.758, df = 9, *p* = 0.764); however, the validity of this test is limited by the presence of multiple cells with expected counts below 5, particularly in the >60 and extremely anxious categories.

An initial ordinal logistic regression model (Model 1), including sex, age group, and chronic disease status as predictors of anxiety level, yielded complementary findings. In this model, female sex showed a trend toward higher anxiety (Model 1: OR = 1.69, 95% CI: 0.98–2.91, *p* = 0.060), while the presence of chronic medical conditions was significantly associated with increased anxiety (Model 1: OR = 3.47, 95% CI: 1.11–10.79, *p* = 0.032). Age group was not a significant predictor (Model 1: OR = 0.92, 95% CI: 0.68–1.25, *p* = 0.605). The corresponding fully adjusted estimates obtained after the addition of visit frequency (Model 2: female sex OR = 1.80, *p* = 0.037; chronic disease OR = 3.84, *p* = 0.022).

### 3.3. Anxiety Symptoms and Triggering Procedures

**Figure 2:**
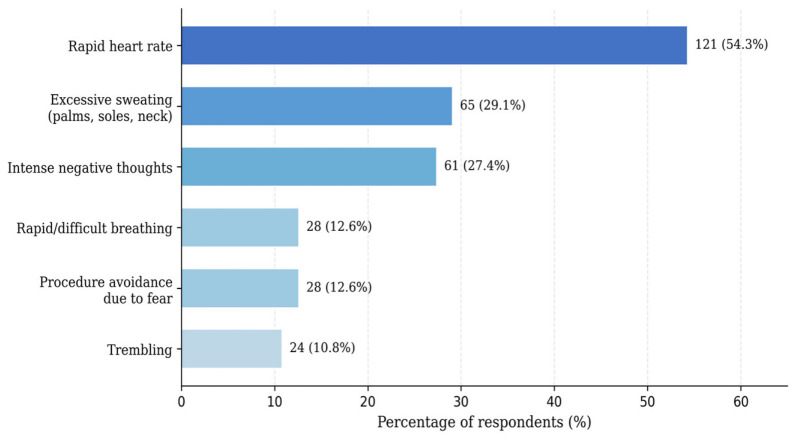
Frequency of self-reported anxiety symptoms experienced before or during dental procedures (*n* = 223). Multiple responses were permitted. Values indicate the number of respondents and the corresponding percentage.

Participants were asked to indicate all anxiety symptoms they typically experienced before or during a dental procedure (Q6; multiple responses permitted). The frequency distribution of reported symptoms is illustrated in Figure 2. The most commonly endorsed symptom was rapid heart rate, reported by over half of the respondents (*n* = 121; 54.3%). Excessive sweating (palms, soles, and neck) was the second most prevalent symptom (*n* = 65; 29.1%), followed closely by intense negative thoughts (*n* = 61; 27.4%). Rapid or difficult breathing and procedure avoidance due to fear were each reported by 12.6% of participants (*n* = 28 each), while trembling was endorsed by 10.8% (*n* = 24). On average, respondents reported 1.76 symptoms (SD = 1.10), with female participants reporting a slightly higher mean number of symptoms (1.90) compared to males (1.44).

Notably, procedure avoidance due to fear, a behavioral indicator consistent with high levels of self-reported dental anxiety, was reported by 12.6% of respondents, a figure that closely mirrors the 11.7% who self-rated their anxiety as very anxious or extremely anxious (Section 3.2). The co-occurrence of autonomic symptoms (rapid heart rate, sweating, rapid breathing) with cognitive symptoms (negative thoughts) and behavioral avoidance in this sample is consistent with the multidimensional nature of dental anxiety described in the literature.

When asked to identify the type of medical procedure that provoked the greatest stress or anxiety (Q7; single-choice), the vast majority of participants selected surgical interventions, including minor surgery (*n* = 142; 63.7%), making this the most anxiety-provoking category by a substantial margin (Table 3, Panel A). Dental procedures were identified as the most stressful by 13.9% of respondents (*n* = 31), followed by blood tests and injections (*n* = 28; 12.6%). Imaging investigations such as MRI or CT (Computed Tomography) scans (*n* = 13; 5.8%) and general medical consultations (*n* = 9; 4.0%) were less commonly perceived as anxiety-provoking.

Among specific dental procedures (Q8; single-choice), tooth extractions (simple or complex) were identified as the most anxiety-provoking by nearly half of the sample (*n* = 111; 49.8%), followed by periodontal surgery (*n* = 88; 39.5%) (Table 3, Panel B). Together, these two invasive categories accounted for 89.3% of all responses, reflecting the strong association between anticipated invasiveness and perceived anxiety. Caries treatment was selected by only 9.4% of respondents (*n* = 21), and professional scaling or cleaning by 1.3% (*n* = 3), indicating that routine, minimally invasive dental procedures elicit comparatively low levels of anticipatory anxiety in this sample.

### 3.4. Anxiety-Reduction Methods Used and Perceived Efficacy

Participants were first asked whether they had previously used any method to reduce anxiety during medical procedures (Q9). The majority of respondents (*n* = 148; 66.4%) reported that they had never used any anxiety-reduction strategy, while approximately one-third (*n* = 64; 28.7%) indicated they had previously used at least one method. A small proportion (*n* = 11; 4.9%) were unsure whether they had used such strategies. These findings suggest that, despite the relatively high prevalence of self-reported anxiety (60.5%), most participants in this sample had not actively adopted any coping mechanism before or during dental procedures. A minor internal inconsistency was observed between Q9 and the subsequent multi-selected item (Q10): although 66.4% reported never having used a method in Q9, a slightly smaller proportion (55.2%) selected the “none” option in Q10. Individual-level cross-tabulation indicated that this gap was almost entirely attributable to respondents who answered “No” (*n* = 30) or “Not sure” (*n* = 7) in Q9 but nonetheless endorsed at least one specific method in Q10—an artefact of the conditional (“if yes”) framing of Q10 not being technically enforced within the online form. As Q9 was a direct, unconditional question, it is treated here as the more reliable estimate of overall prior method use; this limitation is further noted in Section 4.7.

**Figure 3:**
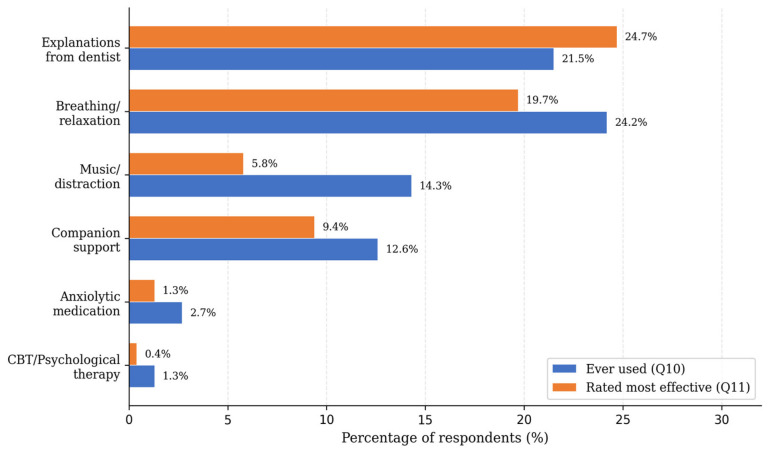
Comparison of anxiety-reduction methods previously used (Q10, blue bars) and methods rated as most effective (Q11, orange bars) among study participants (*n* = 223). “None” category excluded for clarity. Note the discrepancy for music/distraction (higher usage, lower effectiveness rating) and for explanations from the dentist (higher effectiveness rating than usage).

Among those who reported using anxiety-reduction methods or could identify strategies they had employed (Q10; multiple responses permitted), the most frequently cited approaches were breathing and relaxation techniques (*n* = 54; 24.2% of the total sample) and detailed explanations provided by the dentist (*n* = 48; 21.5%) (Table 4; Figure 3). Music listening or distraction was reported by 14.3% (*n* = 32), and companion support from a family member or friend by 12.6% (*n* = 28). Pharmacological strategies were considerably less common: only 6 participants (2.7%) reported using anxiolytic medication prescribed by a physician, and 3 (1.3%) indicated having undergone cognitive-behavioral therapy before a dental procedure. A total of 124 respondents (55.6%) explicitly selected “none” for this question, a figure consistent with the 66.4% who reported in Q9 not having used any method.

When asked to identify the single most effective method for reducing their anxiety (Q11; single-choice), the pattern of responses revealed a notable discrepancy relative to actual usage (Table 4; Figure 3). Detailed explanations from the dentist were rated as the most effective strategy by the largest proportion of respondents who endorsed a specific method (*n* = 55; 24.7%), even though they were only the second-most-commonly used approach (21.5%). Breathing and relaxation techniques, the most frequently used method (24.2%), were rated as most effective by 19.7% of participants (*n* = 44). The presence of a trusted companion was considered most effective by 9.4% (*n* = 21), while music listening or distraction, used by 14.3%, was rated as most effective by only 5.8% (*n* = 13), suggesting that this method, while accessible, may have lower perceived efficacy than other strategies. Anxiolytic medication (*n* = 3; 1.3%) and cognitive-behavioral therapy (*n* = 1; 0.4%) were rated as most effective by very few respondents. A substantial proportion (*n* = 86; 38.6%) selected “none” as most effective, indicating either that they had not used any method or that none of the methods they had tried was considered particularly helpful.

The comparison between actual use and perceived effectiveness across methods reveals several clinically relevant patterns (Figure 3). Detailed explanations from the dentist exhibited a positive perceived-efficacy surplus, being rated as most effective more often than it was reported as used (+3.2 percentage points), suggesting that when provided, this strategy is perceived as highly beneficial by patients. Conversely, music listening or distraction demonstrated the largest perceived-efficacy deficit (−8.5 percentage points), indicating that although relatively many patients had tried it, fewer considered it the single most effective approach. Breathing and relaxation techniques showed a modest deficit (−4.5 points), while companion support was closely aligned between usage and perceived effectiveness (−3.2 points). These patterns suggest that communication-based strategies may yield higher patient-perceived value relative to sensory distraction methods in the dental setting.

### 3.5. Perception of Healthcare Staff Attitude

Participants’ perceptions of the attitude of healthcare staff toward managing their anxiety are summarized in Table 5. The large majority of respondents (*n* = 165; 74.0%) characterized the attitude of dental staff as very empathic and supportive. Approximately one quarter (*n* = 53; 23.8%) perceived the staff as professional but emotionally distant, while a small minority (*n* = 5; 2.2%) described the staff attitude as inappropriate, reporting that no attempt was made to address their anxiety. These findings indicate that, in the experience of most participants, dental practitioners demonstrated a degree of empathic engagement, although nearly one in four patients perceived a relational gap between clinical competence and emotional attunement.

A statistically significant association was observed between pre-procedural anxiety level and perception of staff attitude (χ^2^ = 12.772, df = 2, *p* = 0.002). Among participants who reported any degree of anxiety (*n* = 135), 66.7% rated staff as empathic, compared to 85.2% of those who reported feeling completely relaxed. This pattern suggests that patients with higher anxiety levels may be more likely to perceive staff behavior as emotionally distant or inadequate, although the cross-sectional design precludes determination of the causal direction of this association. The relationship between sex and perceived staff attitude did not reach statistical significance (χ^2^ = 5.255, df = 2, *p* = 0.072), though a trend was observed, with all five respondents who rated staff as inappropriate being female.

When asked to identify the single most helpful strategy used by healthcare staff to reduce their anxiety (Q13), over half of the participants selected detailed explanations about the procedure (*n* = 116; 52.0%), confirming the central role of informational support in patient-perceived anxiety management (Table 5). The remaining responses were evenly distributed between creating a relaxing environment in the dental office (*n* = 45; 20.2%) and providing emotional support through a calm, empathic demeanor (*n* = 45; 20.2%). Procedural pauses to allow the patient to calm down were considered most helpful by a smaller proportion (*n* = 17; 7.6%). Taken together, these data indicate that communication-based and relational strategies were perceived as considerably more beneficial than procedural modifications.

Regarding the impact of anxiety-reduction strategies on confidence in future dental visits (Q14), the majority of respondents reported a positive effect: 61.9% (*n* = 138) indicated that they felt less anxious than before, while 33.2% (*n* = 74) reported a slight improvement but persisting emotional concerns (Table 5). A minority of participants reported no improvement whatsoever: 4.0% (*n* = 9) stated that their anxiety remained unchanged despite the application of strategies, and 0.9% (*n* = 2) reported that they had come to avoid medical procedures as much as possible. Thus, 95.1% of respondents (*n* = 212) reported at least some degree of benefit from the anxiety-reduction approaches they had encountered, suggesting that even modest interventions can contribute to improved patient confidence over time.

A descriptive analysis of the relationship between perceived staff attitude (Q12) and post-strategy confidence (Q14) revealed that among participants who rated staff as very empathic and supportive, 69.1% reported feeling less anxious on future visits, compared to only 39.6% among those who perceived staff as professional but distant. Conversely, 9.4% of those in the “professional but distant” group reported unchanged anxiety, versus only 1.8% in the empathic group. These patterns are consistent with the hypothesis that empathic staff behavior contributes to the cumulative reduction in dental anxiety across visits.

When asked whether they had ever felt that healthcare staff (Q15) did not adequately manage their anxiety, a substantial proportion of respondents endorsed some degree of perceived mismanagement (Table 5). Specifically, 13.0% (*n* = 29) reported feeling that their anxiety was inadequately addressed on multiple occasions, and 29.1% (*n* = 65) reported this experience in certain situations, yielding a combined 42.2% (*n* = 94) who reported at least one instance of perceived inadequate anxiety management. In contrast, 41.7% (*n* = 93) indicated that staff were usually attentive to their anxiety, and 16.1% (*n* = 36) reported not having experienced anxiety during medical procedures. The association between pre-procedural anxiety level and perceived mismanagement was highly significant (χ^2^ = 22.406, df = 3, *p* < 0.001), with anxious participants substantially more likely to report instances of inadequate management than relaxed participants. This finding highlights a potential gap between patient needs and clinical practice in anxiety management, particularly for patients with elevated baseline anxiety.

### 3.6. Patient Preferences for Improvement

Participants were asked to identify the single most important improvement that should be implemented to help anxious patients during medical procedures (Q16; single-choice). The results are presented in Table 6 (Panel A). The most frequently endorsed recommendation was more empathy and communication from clinicians, selected by 40.8% of respondents (*n* = 91). More detailed explanations about procedures before their execution were the second most common preference (*n* = 68; 30.5%), followed by the creation of a more relaxing environment in dental offices (*n* = 47; 21.1%). Offering relaxation techniques before and during the procedure was selected by a smaller proportion (*n* = 17; 7.6%). These findings indicate that the majority of patients prioritize interpersonal and communicative dimensions of care over environmental or technique-based modifications.

**Figure 4:**
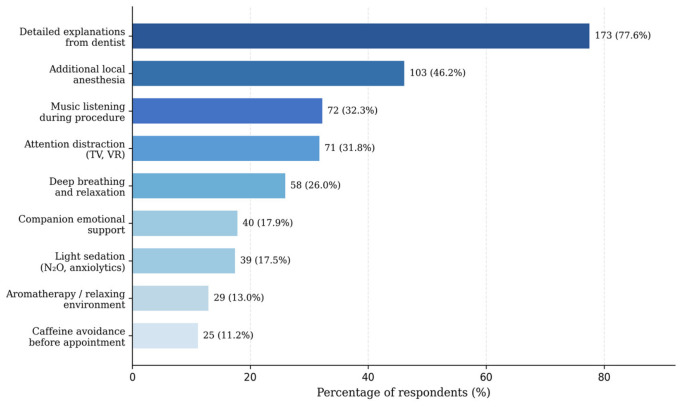
Strategies considered most effective for reducing anxiety during dental procedures (Q17; multiple responses permitted, *n* = 223). Bars represent the number of respondents and the corresponding percentage who endorsed each strategy. Strategies are ranked in descending order of endorsement frequency.

When presented with a comprehensive list of nine anxiety-reduction strategies and asked to select all those they considered most effective for reducing anxiety during dental procedures (Q17; multiple responses permitted), participants endorsed a mean of 3.23 strategies (SD = 1.97), indicating a preference for multimodal approaches to anxiety management. The ranked distribution of endorsed strategies is illustrated in Figure 4.

Detailed explanations provided by the dentist before the procedure emerged as the dominant strategy, endorsed by over three-quarters of respondents (*n* = 173; 77.6%). The second most frequently selected strategy was the use of additional local anesthesia to reduce pain (*n* = 103; 46.2%), reflecting the close conceptual link between anticipated pain and anticipatory anxiety in patients’ perceptions. Music listening during the procedure (*n* = 72; 32.3%) and attention distraction through technology such as television or virtual reality (*n* = 71; 31.8%) were endorsed at comparable rates, suggesting that sensory distraction modalities are valued by approximately one third of respondents. Deep breathing and relaxation techniques were considered effective by 26.0% (*n* = 58). Companion emotional support (*n* = 40; 17.9%) and light sedation with nitrous oxide or anxiolytic medication (*n* = 39; 17.5%) were endorsed at similar, lower frequencies. Aromatherapy or a relaxing office environment (*n* = 29; 13.0%) and caffeine avoidance before the appointment (*n* = 25; 11.2%) were the least frequently selected options.

A descriptive stratification of Q17 responses by anxiety status revealed notable differences in strategy preferences between anxious and relaxed participants. Anxious respondents showed a stronger preference for additional local anesthesia (50.4% vs. 39.8%), attention distraction (36.3% vs. 25.0%), light sedation (23.7% vs. 8.0%), and companion support (20.0% vs. 14.8%) compared to their relaxed counterparts. The most pronounced difference was observed for light sedation, which was endorsed nearly three times more frequently by anxious participants, suggesting that patients with elevated anxiety perceive pharmacological approaches as a necessary complement to non-pharmacological strategies. In contrast, detailed explanations from the dentist were endorsed at comparably high rates by both groups (75.6% anxious vs. 80.7% relaxed), confirming the universal appeal of communication-based interventions regardless of anxiety severity.

The final question (Q18; single-choice) asked participants how the dental care experience could be specifically improved regarding anxiety management. The results (Table 6, Panel B) strongly reinforced the findings from Q16 and Q17: clearer, more detailed communication between clinician and patient before and during the procedure was the most frequently endorsed recommendation (*n* = 120; 53.8%), followed by creating a more relaxing atmosphere in the dental office (*n* = 35; 15.7%). The remaining responses were distributed across several categories at comparatively low frequencies: offering anxiety management options such as breathing techniques or mindfulness (*n* = 15; 6.7%), availability of distraction methods (*n* = 15; 6.7%), application of light sedation for severely anxious patients (*n* = 15; 6.7%), and training of dental staff in anxiety management (*n* = 13; 5.8%). Improvements to anesthesia administration techniques (*n* = 4; 1.8%), flexible scheduling (*n* = 3; 1.3%), and psychological counseling for patients with severe dental anxiety (*n* = 3; 1.3%) were endorsed by very few respondents.

Across all three preference-related questions (Q16, Q17, Q18), a consistent hierarchy of patient priorities emerges. Communication-based strategies, encompassing both empathic interaction and detailed procedural information, constitute the dominant preference in this sample, endorsed as the primary recommendation by 40.8–77.6% of respondents depending on the question format. Pain-reduction strategies, particularly additional local anesthesia, represent a secondary but important priority (46.2%). Sensory distraction modalities (music, VR, television) occupy an intermediate position (31–32%), while pharmacological sedation and environmental modifications are valued by a smaller, though clinically relevant, subset of patients. These findings suggest that improving dentist–patient communication alone could address the primary anxiety management concern of the majority of patients in this sample.

### 3.7. Bivariate Associations and Multivariable Analysis

To explore potential associations between demographic and clinical variables and key study outcomes, a series of Pearson’s chi-square tests was conducted. The results of all bivariate analyses are summarized in Table 7. Where expected cell counts fell below 5, results are reported with appropriate caution, and Fisher’s exact test was employed for 2 × 2 tables. Statistical significance was set at *p* < 0.05.

None of the four demographic variables examined showed a statistically significant association with the four-category anxiety level distribution. The association between sex and anxiety level approached but did not reach significance (χ^2^ = 5.977, df = 3, *p* = 0.113), primarily because the small counts in the extreme anxiety categories limited statistical power. However, when anxiety was dichotomized into moderate-to-severe (very anxious or extremely anxious, *n* = 26) versus low or no anxiety (*n* = 197), sex was significantly associated with moderate-to-severe anxiety (χ^2^ = 4.393, df = 1, *p* = 0.036; Fisher’s exact test *p* = 0.023), with women exhibiting a higher risk (15.0%) compared to men (4.3%). Age group (χ^2^ = 5.758, df = 9, *p* = 0.764), chronic disease status (χ^2^ = 8.947, df = 6, *p* = 0.177), and visit frequency (χ^2^ = 10.934, df = 9, *p* = 0.280) were not significantly associated with the four-category anxiety distribution. These non-significant results should be interpreted with caution, as over 50% of expected cell counts were below 5 in the age, chronic disease, and visit frequency analyses.

Highly significant associations were observed between anxiety status and patient perceptions of care. Participants who reported any degree of pre-procedural anxiety were significantly more likely to perceive staff attitude as distant or inappropriate compared to relaxed participants (χ^2^ = 12.772, df = 2, *p* = 0.002). The strongest association identified in the study was between anxiety status and perceived inadequacy of anxiety management (χ^2^ = 22.406, df = 3, *p* < 0.001), confirming that anxious patients were substantially more likely to report instances in which dental staff did not appropriately address their anxiety. The association between sex and perceived staff attitude showed a trend but did not reach significance (χ^2^ = 5.255, df = 2, *p* = 0.072).

Several significant associations emerged regarding the prior use of anxiety-reduction methods (Q9). Age group was significantly associated with reported method use (χ^2^ = 23.891, df = 6, *p* < 0.001), with older age groups more likely to report having previously employed coping strategies. Chronic disease status was also significantly associated with prior method use (χ^2^ = 18.670, df = 4, *p* < 0.001), suggesting that patients with comorbid chronic conditions may be more experienced with or more initiative-taking about anxiety management. Visit frequency showed a borderline association with method use (χ^2^ = 12.340, df = 6, *p* = 0.055), while sex was not associated (χ^2^ = 0.891, df = 2, *p* = 0.641). When the Bonferroni-corrected threshold (α = 0.0042) is applied, the associations between anxiety status and perceived staff attitude (*p* = 0.002), anxiety status and perceived mismanagement (*p* < 0.001), age group and prior method use (*p* < 0.001), and chronic disease and prior method use (*p* < 0.001) remain statistically significant. In contrast, the borderline association between sex and moderate-to-severe anxiety (*p* = 0.036) and, in the regression analysis, the effect of female sex in Model 2 (*p* = 0.037) would not survive correction for multiple comparisons and should therefore be regarded as preliminary.

To assess the independent effects of demographic and clinical variables on anxiety severity while controlling for potential confounders, two ordinal logistic regression models were fitted using a proportional odds (cumulative logit) framework, with the four-level anxiety variable as the dependent variable (Table 8).

Model 1 included sex, age group (ordinal), and chronic disease status as predictors. Chronic disease was the only statistically significant predictor (OR = 3.47, 95% CI: 1.11–10.79, *p* = 0.032), indicating that participants with chronic medical conditions had approximately 3.5 times the odds of reporting a higher anxiety category compared to those without chronic conditions, after adjusting for sex and age. Female sex showed a trend toward higher anxiety (OR = 1.69, 95% CI: 0.98–2.91, *p* = 0.060) that approached but did not reach conventional significance. Age group was not a significant predictor (OR = 0.92, 95% CI: 0.68–1.25, *p* = 0.605).

Model 2 extended Model 1 by adding visit frequency as a covariate. In this adjusted model, female sex became a statistically significant predictor of higher anxiety (OR = 1.80, 95% CI: 1.04–3.14, *p* = 0.037), and the effect of chronic disease status was strengthened (OR = 3.84, 95% CI: 1.21–12.20, *p* = 0.022); the breadth of this confidence interval, reflecting the small number of participants with chronic conditions (*n* = 13), signals limited precision and potential instability of this estimate. Visit frequency showed a trend toward a protective association, with more frequent dental attendance associated with lower anxiety, although this did not reach significance (OR = 0.75, 95% CI: 0.53–1.05, *p* = 0.092). Age group remained non-significant (OR = 0.89, 95% CI: 0.66–1.22, *p* = 0.478). Model 2 showed marginally improved fit (AIC = 463.2) compared to Model 1 (AIC = 464.1), though the difference was small.

In summary, and female sex as statistically significant independent predictors of dental anxiety severity in this sample, the latter reaching significance only after adjustment for visit frequency. The chronic disease estimate, however, was based on only 13 participants with confirmed chronic conditions and was accompanied by a very wide confidence interval (OR = 3.84, 95% CI: 1.21–12.20), indicating substantial imprecision; this association should therefore be regarded as unstable and interpreted with considerable caution despite reaching nominal significance. The absence of a significant age effect contrasts with some international findings but is consistent with the limited age variability in this predominantly young sample. The borderline protective trend for visit frequency is consistent with the hypothesis that regular dental attendance may contribute to desensitization and reduced anticipatory anxiety over time.

## 4. Discussion

### 4.1. Main Findings Summary

The present cross-sectional survey of 223 Romanian adults generated a comprehensive, patient-centered profile of self-reported dental anxiety, encompassing prevalence, symptomatology, use of coping strategies, perceptions of healthcare staff, and preferences for clinical improvement. The following subsections interpret the principal findings in the context of the existing international literature and discuss their implications for dental practice and education.

### 4.2. Comparison with International Literature

Direct comparison with international meta-analytic data requires careful attention to differing anxiety thresholds. Silveira et al. (2021), encompassing over 72,000 adults, estimated the global prevalence of high dental anxiety at 12.4% (95% CI: 8.0–17.7%) and of severe dental anxiety at 3.3% [1]; these figures are closely approximated by the present study’s moderate-to-severe (11.7%) and extreme anxiety (2.7%) categories, respectively, obtained using comparable severity thresholds. In contrast, the broader figure of 60.5% reflects any self-reported degree of anxiety, including mild or slight worry, and is not directly comparable to the global high/severe prevalence estimate of 15.3% reported by Silveira et al. This broader threshold is more appropriately compared with studies employing similarly low cut-offs; for example, Peric and Tadin (2024), in a large Croatian online survey (*n* = 1240), reported that nearly two-thirds of respondents acknowledged some degree of dental anxiety [3], a figure consistent with the present 60.5% estimate. The somewhat lower prevalence of severe anxiety in the present sample may reflect the predominance of younger, healthy individuals recruited via online convenience sampling, which likely underrepresented populations with heightened dental anxiety.

It should be noted that direct numerical comparison between the present findings and studies employing validated instruments is limited by the use of a non-validated four-point ordinal self-report item in the present investigation (see Section 4.7). Nevertheless, the broad concordance between our estimates and those from MDAS-based studies suggests that in self-reported anxiety severity. Among Romanian studies, Zegan et al. (2019) reported high dental anxiety in 4.3% and severe anxiety in 2.9% [35] using the DAS-R and MDAS among 210 patients from northeastern Romania [15,17,22], while Armencia et al. (2025) found that 6.1% of 180 dental students from Iași exhibited severe anxiety [36]. The psychosocial dimension of dental encounters has also been explored in the context of COVID-19, with Moroianu et al. (2026) demonstrating that dental emergencies carried a significant psychosocial impact in affected patients, consistent with the broader vulnerability to anxiety observed in medically compromised populations [37]. Differences across these studies are attributable to variations in instruments, recruitment settings, and sample composition.

The significantly higher prevalence of moderate-to-severe anxiety among females (Fisher’s exact *p* = 0.023; OR = 3.96) is consistent with one of the most robustly replicated findings in the dental anxiety literature [1,4]. Proposed explanatory mechanisms include differential pain sensitivity, greater somatic awareness, sex-related differences in trait anxiety, and sociocultural norms permitting greater expression of fear among women [1,4,10]. In the ordinal logistic regression, female sex became statistically significant in Model 2 (OR = 1.80, *p* = 0.037) after adjusting for visit frequency, providing multivariable support for a sex-related vulnerability independent of other demographic factors.

The absence of a significant age effect (*p* = 0.764) is consistent with the limited age variability of the present sample (62.3% aged 18–30 years; only 3.6% over 60 years), which resulted in inadequate statistical power and a high proportion of cells with expected counts below 5. Notably, the small >60 subgroup (*n* = 8) showed the highest nominal proportion of very anxious responses (25.0%), a finding that, if confirmed in age-balanced samples, would be consistent with the hypothesis that older patients with longer histories of potentially aversive dental experiences may accumulate higher levels of procedural anxiety [1,5,6,17].

The association between chronic medical conditions and anxiety severity (OR = 3.84, *p* = 0.022 in Model 2) is clinically plausible, as patients with comorbidities may experience heightened procedural anxiety due to concerns about safety, drug interactions, or disease exacerbation [10,13]. This observation is consistent with broader clinical evidence from Romanian hospital settings demonstrating elevated anxiety and depressive symptom burden among patients with chronic comorbid conditions, including cancer and type 2 diabetes mellitus, where psychiatric morbidity was identified in a substantial proportion of medically assessed inpatients [38,39]. The significantly higher rate of prior coping method uses in this subgroup (χ^2^ = 18.670, *p* < 0.001) suggests that chronic disease patients may possess more elaborate coping repertoires, a bidirectional relationship warranting prospective investigation.

### 4.3. Anxiety Symptoms and Avoidance

Rapid heart rate, the most commonly reported symptom (54.3%), is consistent with the well-characterized pattern of sympathetic activation during anticipatory and procedural stress [10,13]. Hmud and Walsh (2009) described tachycardia as among the most frequently reported physiological manifestations of dental fear, noting its particular clinical relevance in patients with cardiovascular comorbidities [40]. The co-occurrence of autonomic (tachycardia, sweating), cognitive (negative thoughts, 27.4%), and behavioral (avoidance, 12.6%) symptoms reflects the multidimensional structure of dental anxiety operationalized in the IDAF-4C+, validated in Romanian by Done et al. (2023) and in the DFS [16,21].

Procedure avoidance due to fear (12.6%) closely mirrors the proportion reporting moderate-to-severe anxiety on the self-report scale (11.7%), suggesting reasonable internal consistency between behavioral and subjective dimensions. This convergence is clinically significant, as avoidance represents the central mechanism through which dental anxiety translates into oral health deterioration via Berggren’s vicious cycle [5,6]. Armfield (2013) demonstrated that individuals with high dental fear were significantly more likely to delay treatment and exhibit symptomatic visiting patterns [6], while Aardal et al. (2023) showed that the behavioral avoidance component was the strongest predictor of impaired oral health-related quality of life, independently of other anxiety dimensions [9]. These findings underscore the need for proactive identification of avoidant patients through standardized screening before treatment planning [10,13,16,21,40].

Regarding anxiety-provoking procedures, the dominance of tooth extractions (49.8%) and periodontal surgery (39.5%), together accounting for 89.3% of responses, reflects the well-established association between anticipated invasiveness and anticipatory anxiety [10,13].

### 4.4. Patient-Preferred Strategies

The most prominent finding of the present study is the consistent endorsement of detailed procedural explanations as the dominant patient-preferred anxiety-reduction strategy across four independent survey items employing different response formats: 77.6% endorsed clinician explanations in Q17, 52.0% identified them as the most helpful staff strategy (Q13), 24.7% rated them as the most effective method experienced (Q11), and 53.8% recommended clearer communication as the primary improvement to dental care (Q18). This convergence provides robust evidence that informational and communicative support constitutes the primary patient-centered priority, well above pharmacological, environmental, or distraction-based alternatives.

This finding aligns with the “tell-show-do” framework and is theoretically grounded in the established relationship between perceived control and anxiety: uncertainty about an impending aversive event amplifies anticipatory fear, and accurate procedural information reduces unpredictability [10,13,25]. Appukuttan (2016) identified empathic communication as the foundational non-pharmacological strategy, noting that adequately informed patients consistently report lower anxiety and greater willingness to attend future appointments [10]. Yuan et al. (2020) provided empirical evidence that communication quality and trust were independently associated with dental anxiety levels and attendance patterns [26].

The comparison between actual use and perceived effectiveness revealed clinically relevant discrepancies. Detailed explanations exhibited a positive “perceived-efficacy surplus,” being rated as most effective more often than actually used (+3.2 percentage points), indicating high patient-perceived value when provided. In contrast, music listening and distraction demonstrated the largest “perceived-efficacy deficit” (−8.5 percentage points), suggesting that while conceptually valued, their perceived effectiveness among respondents may be more modest than anticipated. This interpretation is consistent with the current evidence base: Steenen et al. (2024) concluded that pooled evidence does not yet conclusively support music as an effective stand-alone intervention for dental anxiety, with considerable heterogeneity attributable to variability in music type, delivery mode, and clinical context [27,29,31].

Additional local anesthesia (endorsed by 46.2%) emerged as the second most preferred strategy, reflecting the intimate conceptual link between anticipated pain and anticipatory anxiety [5,6,10]. Anxious respondents endorsed this at a higher rate than relaxed counterparts (50.4% vs. 39.8%), consistent with documented associations between elevated anxiety and lower pain thresholds [13,40]. Light sedation was endorsed by 17.5% overall but 23.7% among anxious participants versus 8.0% among relaxed respondents, the most pronounced anxiety-stratified difference across all strategies, indicating that pharmacological sedation is perceived as a necessary complement by a clinically relevant subgroup. This pattern supports a stepped-care model in which communication-based strategies serve as the universal foundation, with pharmacological options available for patients with more severe anxiety, consistent with the frameworks described by Appukuttan (2016), Hoffmann et al. (2022), and Steenen et al. (2024) [10,13,27].

### 4.5. Role of Dental Staff in Anxiety Management

Although 74.0% of respondents characterized staff attitude as very empathic and supportive, a combined 42.2% reported at least one instance of perceived inadequate anxiety management, and 23.8% perceived staff as professional but emotionally distant. A significant association was observed between anxiety status and perceived staff attitude (χ^2^ = 12.772, *p* = 0.002), and the strongest association in the entire study was between anxiety status and perceived inadequacy of anxiety management (χ^2^ = 22.406, *p* < 0.001), confirming that anxious patients represent the subgroup with the greatest unmet need for structured anxiety management.

These findings are consistent with the hypothesis that patients with elevated anxiety are more sensitive to relational cues during clinical encounters and more attentive to perceived deficits in emotional attunement [10,26]. This observation is consistent with findings from pediatric psychiatric consultations, where physician empathy as perceived by patients or their caregivers was shown to significantly influence the quality and outcome of the clinical encounter [41], suggesting that empathic communication constitutes a transdiagnostic and cross-specialty determinant of patient experience. However, the cross-sectional design precludes the determination of causal direction: genuinely inadequate staff behavior may contribute to the maintenance of anxiety, anxious patients may be more critical evaluators, or both mechanisms may operate simultaneously.

The positive impact of anxiety-management strategies on confidence in future dental visits, reported by 95.1% of respondents, provides preliminary patient-reported evidence that even modest interventions can produce cumulative benefits. Among patients who perceived staff as empathic, 69.1% reported feeling substantially less anxious before future visits, compared to 39.6% among those perceiving distant staff. This pattern is consistent with the hypothesis that empathic staff behavior contributes to the progressive habituation of dental anxiety across successive visits, potentially disrupting Berggren’s vicious cycle [5,6], Bernson et al. (2011) demonstrated that patients reporting positive interactions with dental staff were significantly more likely to maintain regular attendance despite elevated trait anxiety [42] while Hoffmann et al. (2022) identified communication-based approaches as the most broadly applicable non-pharmacological strategy [10,13].

### 4.6. Implications for Clinical Practice

The present findings carry several direct implications for dental practice.

These implications are derived from patient-reported preferences obtained in a cross-sectional, convenience-sampled survey using a non-validated instrument; they should therefore be read as preference-informed suggestions to be weighed alongside existing controlled evidence, rather than as efficacy-based recommendations established by the present data.

**Systematic anxiety screening**. The high prevalence of self-reported dental anxiety (60.5%) and the significant proportion reporting perceived inadequate management (42.2%) argue for the routine implementation of brief validated screening instruments. The MDAS, requiring less than two minutes to complete and validated in Romanian [15,17,22], or the IDAF-4C+, offering a multidimensional assessment validated in Romanian by Done et al. (2023) [21], could be incorporated into standard intake procedures with minimal disruption to clinical workflows.

**Prioritizing clinician–patient communication**. The consistent patient endorsement of detailed procedural explanations, considered alongside existing controlled evidence, supports positioning proactive clinician–patient communication as a foundational element of anxiety-sensitive care. These data suggest that offering structured pre-procedural information in plain language, together with an explicit invitation to express concerns, may be a reasonable default approach for anxious patients, although our cross-sectional design and non-validated instrument preclude any causal claim regarding its effectiveness [13,25]. Critically, the present data distinguish between informationally complete but affectively neutral communication (“professional but distant”) and genuinely empathic communication that validates the patient’s emotional state, the latter being associated with substantially higher post-strategy confidence [10,26].

Proactive offering of non-pharmacological strategies. The finding that 66.4% of respondents had never used any anxiety-reduction method despite 60.5% reporting anxiety reveals a gap that likely reflects insufficient proactive clinical offering rather than a lack of patient need. Breathing techniques, music provision, and accommodating companion presence are low-cost interventions that should be systematically offered to all patients identified as anxious may reflect insufficient proactive clinical offering rather than a lack of patient need, although this interpretation remains tentative given the study design. Breathing techniques, music provision, and accommodating companion presence are low-cost interventions that could reasonably be offered to patients identified as anxious, consistent with existing evidence on their acceptability and potential benefit [10,13]; their effectiveness in this population would, however, need to be confirmed in prospective controlled studies.

**Stepped-care pharmacological provision**. For patients with moderate-to-severe anxiety (11.7%), nitrous oxide inhalation sedation represents the first pharmacological step, followed by oral benzodiazepine premedication and intravenous conscious sedation for patients with more severe needs [13,26,32,40,43].

**Staff training**. The 42.2% reporting inadequately managed anxiety and the 23.8% perceiving distant staff behavior point to a systemic gap addressable through structured communication and empathy training in both undergraduate curricula and postgraduate continuing professional development [10,13,26].

### 4.7. Limitations

Several limitations warrant acknowledgment. First, the cross-sectional design precludes causal inference; the associations identified between demographic variables, anxiety levels, and perceptions of staff attitude cannot be interpreted as directional or causal relationships. Second, convenience sampling via social media introduces selection bias: individuals with extreme dental phobia who tend to avoid dental-related topics are likely underrepresented, potentially leading to underestimation of severe anxiety prevalence. Third, the sample was demographically skewed, with 62.3% of respondents aged 18–30 years and 68.6% female, limiting representativeness and constraining the statistical power of age-stratified analyses. Fourth, the study employed a purpose-designed, non-validated questionnaire rather than a standardized instrument such as the MDAS or IDAF-4C+, which precludes direct score-based comparison with the international literature and leaves the psychometric properties of the individual items empirically unestablished. Fifth, all data were self-reported, introducing possible social desirability bias, recall bias, and overestimation of dental visit frequency. Additionally, effectiveness ratings for anxiety-reduction strategies reflect patients’ subjective perceptions rather than objectively measured clinical efficacy; establishing the latter would require controlled intervention studies. Finally, recruitment was concentrated within the principal investigator’s networks in the Galați region, limiting regional representativeness. These limitations indicate that the present findings should be interpreted as exploratory and hypothesis-generating.

### 4.8. Future Directions

The present study identifies several avenues for future research. Longitudinal prospective studies tracking anxiety levels, coping strategy use, and dental attendance across multiple visits would enable causal inference regarding the factors that predict anxiety reduction or persistence and allow evaluation of the cumulative anxiolytic effects of communication-based strategies. Randomized controlled trials are needed to establish the efficacy of specific anxiety management protocols in Romanian dental settings, where RCT-level evidence is currently absent. The development and psychometric validation of a comprehensive Romanian-language dental anxiety experience questionnaire integrating severity measurement with experiential domains would enhance cross-study comparability. Future studies should employ multicentric, age-stratified sampling to ensure adequate representation of older adults, male patients, rural populations, and individuals with dental phobias. Finally, the integration of objective measures, including heart rate variability, salivary cortisol, and clinical oral health indices, alongside self-report instruments, would strengthen the evidence base and provide health economic arguments for systematic anxiety management investment at the healthcare system level.

## 5. Conclusions

This cross-sectional study of 223 Romanian adult respondents demonstrated that self-reported dental anxiety is a prevalent concern, with 60.5% of participants reporting at least some degree of pre-procedural anxiety and 11.7% endorsing moderate-to-severe levels. Despite this high prevalence, two-thirds of respondents had never actively employed any anxiety-reduction strategy, indicating a substantial gap between clinical need and current management practice. Across all preference-related survey items, detailed procedural explanations provided by the dental clinician emerged as the overwhelmingly dominant patient-preferred strategy (endorsed by 52.0–77.6% of respondents), consistently surpassing pharmacological, technological, and sensory-distraction modalities. Empathic staff communication was independently associated with reduced perceived anxiety and greater patient confidence. These findings carry direct implications for dental education curricula and clinical practice: structured communication training for dental staff, integration of standardized anxiety screening into routine workflows, and the adoption of stepped-care protocols that prioritize informational and relational strategies before pharmacological interventions could meaningfully improve the dental care experience for anxious patients.

## Figures and Tables

**Table 1:** Sociodemographic characteristics of study participants (*n* = 223).

Variable	Category	*n*	(%)
Sex	Female	153	68.6
	Male	70	31.4
Age group (years)	18–30	139	62.3
	31–45	48	21.5
	46–60	28	12.6
	>60	8	3.6
Chronic medical conditions	Yes	13	5.8
	No	206	92.4
	Not sure	4	1.8
Frequency of dental visits	Very rarely (once every few years)	51	22.9
	Once per year	106	47.5
	Several times per year (4–6/year)	59	26.5
	Frequently (monthly/weekly)	7	3.1

Note: Percentages may not sum to 100% due to rounding. Chronic medical conditions include asthma, HIV/AIDS, hepatitis B or C, hypertension, and diabetes mellitus.

**Table 2:** Distribution of self-reported pre-procedural dental anxiety levels by sex and age group (*n* = 223).

Subgroup	*n* (%)	Completely Relaxed *n* (%)	Slightly Worried *n* (%)	Very Anxious *n* (%)	Extremely Anxious *n* (%)	χ^2^	Degrees of Freedom (df)	*p*
Sex						5.977	3	0.113
Female	153 (68.6)	57 (37.3)	73 (47.7)	17 (11.1)	6 (3.9)			
Male	70 (31.4)	31 (44.3)	36 (51.4)	3 (4.3)	0 (0.0)			
Age group (years)						5.758	9	0.764
18–30	139 (62.3)	52 (37.4)	71 (51.1)	13 (9.4)	3 (2.2)			
31–45	48 (21.5)	23 (47.9)	20 (41.7)	3 (6.2)	2 (4.2)			
46–60	28 (12.6)	10 (35.7)	15 (53.6)	2 (7.1)	1 (3.6)			
>60	8 (3.6)	3 (37.5)	3 (37.5)	2 (25.0)	0 (0.0)			
Total	223 (100)	88 (39.5)	109 (48.9)	20 (9.0)	6 (2.7)			

Note: Values are presented as *n* (row %). χ^2^ = Pearson’s chi-square statistic. Statistical significance was set at *p* < 0.05. For the sex × anxiety analysis, the minimum expected cell count was 1.88; for age × anxiety, several cells had expected counts < 5, and results should be interpreted with caution. When anxiety was dichotomized into moderate-to-severe (very + extremely anxious) versus low/none, a statistically significant sex difference was observed (Fisher’s exact test, *p* = 0.023).

**Table 3:** Most anxiety-provoking medical procedures (Panel A) and dental procedures (Panel B) as reported by study participants (*n* = 223).

Procedure Type	*n*	(%)
Panel A: Most anxiety-provoking medical procedures (Q7)		
Surgical interventions (including minor)	142	63.7
Dental procedures	31	13.9
Blood tests/injections	28	12.6
Imaging investigations (MRI, CT)	13	5.8
General medical consultations	9	4.0
Panel B: Most anxiety-provoking dental procedures (Q8)		
Tooth extractions (simple or complex)	111	49.8
Periodontal surgery	88	39.5
Caries treatment	21	9.4
Professional scaling/cleaning	3	1.3

Note: Both questions were single-choice. Percentages are calculated based on the total sample (*n* = 223). Panel A refers to general medical procedures (Q7); Panel B refers specifically to dental procedures (Q8).

**Table 4:** Anxiety-reduction methods previously used (Q10, multiple responses) and a single method rated as most effective (Q11) by study participants (*n* = 223).

Anxiety-Reduction Method	Ever Used (Q10) *n* (%)	Rated Most Effective (Q11) *n* (%)
Breathing/relaxation techniques	54 (24.2)	44 (19.7)
Detailed explanations from the dentist	48 (21.5)	55 (24.7)
Music listening/distraction	32 (14.3)	13 (5.8)
Companion support (family, friend)	28 (12.6)	21 (9.4)
Anxiolytic medication (benzodiazepines, supplements)	6 (2.7)	3 (1.3)
Cognitive-behavioral therapy	3 (1.3)	1 (0.4)
None	124 (55.6)	86 (38.6)

Note: Q10 permitted multiple responses; percentages exceed 100% when summed. Q11 was single-choice. “None” in Q10 indicates respondents who reported not using any method; “None” in Q11 indicates respondents who did not consider any method most effective. All percentages are based on the total sample (*n* = 223).

**Table 5:** Perception of healthcare staff attitude (Q12), most helpful staff-implemented strategy (Q13), post-intervention confidence (Q14), and perceived adequacy of anxiety management (Q15) among study participants (*n* = 223).

Variable/Response Category	*n*	(%)
Perceived staff attitude toward anxiety management (Q12)		
Very empathic and supportive	165	74.0
Professional but distant	53	23.8
Inappropriate, did not attempt to calm me	5	2.2
Most helpful staff-implemented strategy (Q13)		
Detailed explanations about the procedure	116	52.0
Creating a relaxing environment in the office	45	20.2
Providing emotional support through a calm, empathic demeanor	45	20.2
Pauses during the procedure to allow calming	17	7.6
Post-strategy confidence in future procedures (Q14)		
Yes, I feel less anxious than before	138	61.9
Slightly, but I still have emotions	74	33.2
No, my anxiety has remained the same	9	4.0
No, I have come to avoid procedures as much as possible	2	0.9
Perceived inadequate management of anxiety by staff (Q15)		
Yes, on multiple occasions	29	13.0
Yes, but only in certain situations	65	29.1
No, the staff were usually attentive to my anxiety	93	41.7
I have not experienced anxiety during medical procedures	36	16.1

Note: All questions were single-choice. Percentages are calculated based on the total sample (*n* = 223) and may not sum exactly to 100% due to rounding.

**Table 6:** Patient-recommended improvements for anxiety management in dental practice: general improvements (Q16, Panel A) and specific improvements in dental care (Q18, Panel B) (*n* = 223).

Response Category	*n*	(%)
General improvements needed in dental practice (Q16)		
More empathy and communication from clinicians	91	40.8
More detailed explanations before procedures	68	30.5
Creating a more relaxing environment in dental offices	47	21.1
Offering relaxation techniques before and during the procedure	17	7.6
Specific improvements in dental care regarding anxiety (Q18)		
Clearer, more detailed communication before and during the procedure	120	53.8
Creating a more relaxing atmosphere in the office	35	15.7
Offering anxiety management options (breathing, mindfulness, step-by-step explanations)	15	6.7
Availability of distraction methods (TV, VR, personalized music)	15	6.7
Application of light sedation techniques for severely anxious patients	15	6.7
Training dental staff in patient anxiety management	13	5.8
Improving anesthesia administration techniques	4	1.8
Flexible scheduling to reduce waiting time and anticipatory stress	3	1.3
Psychological support/counseling for patients with severe dental anxiety	3	1.3

Note: Both Q16 and Q18 were single-choice questions. Percentages are based on the total sample (*n* = 223). Q17 (multi-select) data are presented in Figure 4.

**Table 7:** Summary of bivariate chi-square analyses between demographic/clinical variables and key study outcomes (*n* = 223).

Independent Variable	Dependent Variable	χ^2^	df	*p*-Value	Min. Exp.	Cells < 5 (%)
Sex	Anxiety level (4-cat)	5.977	3	0.113	1.88	25
Sex	Moderate-severe anxiety	4.393 ^b^	1	0.036 *	8.16	0
Age group	Anxiety level (4-cat)	5.758	9	0.764	0.22	56
Chronic disease	Anxiety level (4-cat)	8.947	6	0.177	0.11	50
Visit frequency	Anxiety level (4-cat)	10.934	9	0.280	0.19	50
Sex	Staff attitude (Q12)	5.255	2	0.072	1.57	33
Anxiety (binary)	Staff attitude (Q12)	12.772	2	0.002 *	1.97	33
Anxiety (binary)	Mismanagement (Q15)	22.406	3	<0.001 *	11.44	0
Sex	Prior method use (Q9)	0.891	2	0.641	3.45	17
Visit frequency	Prior method use (Q9)	12.340	6	0.055	0.35	42
Age group	Prior method use (Q9)	23.891	6	<0.001 *	0.39	33
Chronic disease	Prior method use (Q9)	18.670	4	<0.001 *	0.20	56

Note: χ^2^ = Pearson’s chi-square statistic. * *p* < 0.05. Min. exp. = minimum expected cell count. Cells < 5 = percentage of cells with expected count below 5. Moderate-to-severe anxiety = very anxious + extremely anxious vs. all others. ^b^ Yates-corrected chi-square; Fisher’s exact test yielded *p* = 0.023, OR = 3.96. Results with >20% of cells having expected counts < 5 should be interpreted with caution.

**Table 8:** Ordinal logistic regression (proportional odds model) predicting self-reported dental anxiety severity (4 levels) from demographic and clinical variables (*n* = 223).

Predictor	Coeff. (β)	SE	OR	95% CI	*p*-Value
Model 1 (AIC = 464.1)					
Female sex (vs. male)	0.523	0.278	1.687	0.978–2.908	0.060
Age group (ordinal)	−0.081	0.156	0.923	0.680–1.252	0.605
Chronic disease (yes vs. no)	1.243	0.580	3.465	1.113–10.791	0.032 *
Model 2 (AIC = 463.2)					
Female sex (vs. male)	0.590	0.282	1.803	1.036–3.137	0.037 *
Age group (ordinal)	−0.112	0.158	0.894	0.656–1.218	0.478
Chronic disease (yes vs. no)	1.346	0.590	3.842	1.210–12.203	0.022 *
Visit frequency (ordinal)	−0.290	0.172	0.748	0.534–1.049	0.092

Note: OR = proportional odds ratio; 95% CI = 95% confidence interval; SE = standard error; AIC = Akaike Information Criterion. * *p* < 0.05. The dependent variable is the four-level ordinal anxiety scale (completely relaxed/slightly worried/very anxious/extremely anxious). ORs > 1 indicate higher odds of being in a more severe anxiety category. Model 1 includes sex, age group, and chronic disease. Model 2 adds visit frequency. Both models were estimated using maximum likelihood with the BFGS algorithm. The proportional odds assumption was supported by the Brant test for both models (Model 1: omnibus χ^2^ = 3.06, df = 6, *p* = 0.80; Model 2: omnibus χ^2^ = 5.28, df = 8, *p* = 0.73), with no individual predictor showing significant departure from proportionality (all *p* > 0.20). A sensitivity analysis collapsing the two most severe anxiety categories yielded a similarly non-significant result (omnibus χ^2^ = 4.54, df = 4, *p* = 0.34), confirming the validity of the proportional odds specification; multinomial regression was therefore not required.

## Data Availability

The raw data supporting the conclusions of this article will be made available by the authors on request.

## Supplementary Materials

The following supporting information can be downloaded at: https://www.mdpi.com/article/10.3390/dj14070438/s1, File S1: Questionnaire (English translation).

## Abbreviations

The following abbreviations are used in this manuscript:

AIC	Akaike Information Criterion
aOR	Adjusted Odds Ratio
CBT	Cognitive Behavioral Therapy
CEU	Linear dichroism
CI	Confidence Interval
CT	Computed Tomography
DA	Dental Anxiety
DAS	Dental Anxiety Scale
DFS	Dental Fear Survey
df`	Degrees of Freedom
GDPR	General Data Protection Regulation
IDAF-4C+	Index of Dental Anxiety and Fear
MDAS	Modified Dental Anxiety Scale
MRI	Magnetic Resonance Imaging
N	Total number of participants
n	Number of observations in subgroup
OHRQoL	Oral Health-Related Quality of Life
OR	Odds Ratio
RCT	Randomized Controlled Trial
SD	Standard Deviation
STAI	State-Trait Anxiety Inventory
VR	Virtual Reality
χ^2^	Pearson’s Chi-Square Statistic
