# Supplementary material for: Dental Anxiety Prevalence, Correlates, and Patient-Preferred Management Strategies in Romanian Adults: A Cross-Sectional Survey
Source: Dent J (Basel). 2026 Jul 14;14(7):438. doi: 10.3390/dj14070438 (PMC13409583; doi:10.3390/dj14070438)
Supplement: Supplementary file 1 [file dentistry-14-00438-s001.zip › dentistry-4314532-supplementary.pdf]

Supplementary File S1: Questionnaire (English translation)

1. Sex

a) Male

b) Female

2. Age

a) 18–30 years

b) 31–45 years

c) 46–60 years

d) Over 60 years

3. Do you have any chronic medical conditions? (e.g., asthma, HIV/AIDS, hepatitis B, hepatitis C, hypertension, diabetes mellitus)

a) Yes

b) No

c) I am not sure

4. How often do you attend medical appointments?

a) Very rarely (once every few years)

b) Once a year

c) Several times a year (4–6 times)

d) Frequently (monthly/weekly)

5. How do you generally feel before a dental procedure?

a) Completely relaxed

b) Slightly worried

c) Very anxious

d) Extremely anxious; I sometimes avoid dental procedures

6. Which anxiety symptoms do you experience most frequently before or during a medical procedure? (Select all that apply.)

a) Increased heart rate

b) Excessive sweating (palms, soles, neck)

c) Trembling

d) Rapid breathing or difficulty breathing

e) Intense negative thoughts

f) Avoiding the procedure because of fear

7. Which of the following medical procedures causes you the greatest stress or anxiety?

a) Blood tests/injections

b) Dental procedures

c) Surgical procedures (including minor surgery)

d) Imaging investigations (e.g., MRI, CT scan)

e) General medical consultations

8. Which dental procedure causes you the greatest stress or anxiety?

a) Simple or surgical tooth extraction

b) Periodontal surgery

c) Dental caries treatment (fillings)

d) Professional dental cleaning

9. Have you previously used any methods to reduce anxiety during medical procedures?

a) Yes

b) No

c) I am not sure

10. If yes, which method(s) have you used? (Select all that apply.)

a) Breathing or relaxation techniques

b) Listening to music/distraction

c) Cognitive-behavioral therapy before the procedure (long-term)

d) Support from a companion (family member or friend)

e) Detailed explanations provided by the physician

f) Physician-prescribed anxiolytic medication (e.g., benzodiazepines or calming supplements)

g) None

11. Which of the above methods was the most effective in reducing your anxiety?

a) Breathing/relaxation techniques

b) Listening to music/distraction

c) Psychological therapy before the procedure

d) Presence of a trusted person

e) Explanations provided by the physician

f) Anxiolytic medication

12. How would you describe the attitude of the healthcare staff in helping reduce your anxiety?

a) Very empathetic and supportive

b) Professional but distant

c) Inappropriate; they did not try to calm me

13. Which strategy used by the healthcare staff helped you feel less anxious?

a) Detailed explanations about the procedure

b) Creating a relaxing environment in the clinic/hospital

c) Taking breaks during the procedure to help me relax

d) Providing emotional support through a calm and empathetic attitude

14. After anxiety-reducing strategies were applied, did you feel more confident about future medical procedures?

a) Yes, I feel less anxious than before

b) Slightly, but I still feel anxious

c) No, my anxiety remained the same

d) No, I have started avoiding medical procedures whenever possible

15. Have you ever felt that your anxiety was not properly managed by healthcare professionals?

a) Yes, on several occasions

b) Yes, but only in certain situations

c) No, healthcare professionals were generally attentive to my anxiety

d) I have never experienced anxiety during medical procedures

16. What improvements do you think should be implemented to help anxious patients during medical procedures?

a) Greater empathy and communication from healthcare professionals

b) More detailed explanations before procedures

c) Creating a more relaxing environment in healthcare facilities

d) Providing relaxation techniques before and during procedures

17. Which of the following strategies do you consider most effective for reducing anxiety during dental procedures? (Select all that apply.)

a) Detailed explanations provided by the dentist before the procedure

b) Deep breathing and relaxation techniques

c) Listening to music during treatment

d) Additional local anesthesia to reduce pain

e) Distraction techniques (e.g., television, virtual reality)

f) Mild sedation (nitrous oxide or anxiolytic medication)

g) Emotional support from a companion

h) Aromatherapy or a relaxing dental office environment

i) Avoiding caffeine before the appointment

18. How do you think anxiety management in the dental office could be improved?

a) Clearer and more detailed communication between the dentist and the patient before and during the procedure

b) Creating a more relaxing atmosphere in the dental office

c) Offering anxiety management options (breathing techniques, mindfulness, step-by-step explanations)

d) Providing distraction methods (television, virtual reality, personalized music)

e) Using mild sedation techniques for patients with severe anxiety

f) Flexible scheduling to reduce waiting time and anticipatory stress

g) Psychological support or counseling for patients with severe dental anxiety

h) Improving anesthesia administration techniques to make procedures more comfortable

i) Training healthcare professionals in anxiety management
